# Supplementary material for: The microRNA miR-71 suppresses maladaptive UPRmt signaling through both cell-autonomous and cell-non-autonomous mechanisms
Source: Nat Commun. 2025 Dec 14;17:510. doi: 10.1038/s41467-025-67198-2 (PMC12804905; doi:10.1038/s41467-025-67198-2)

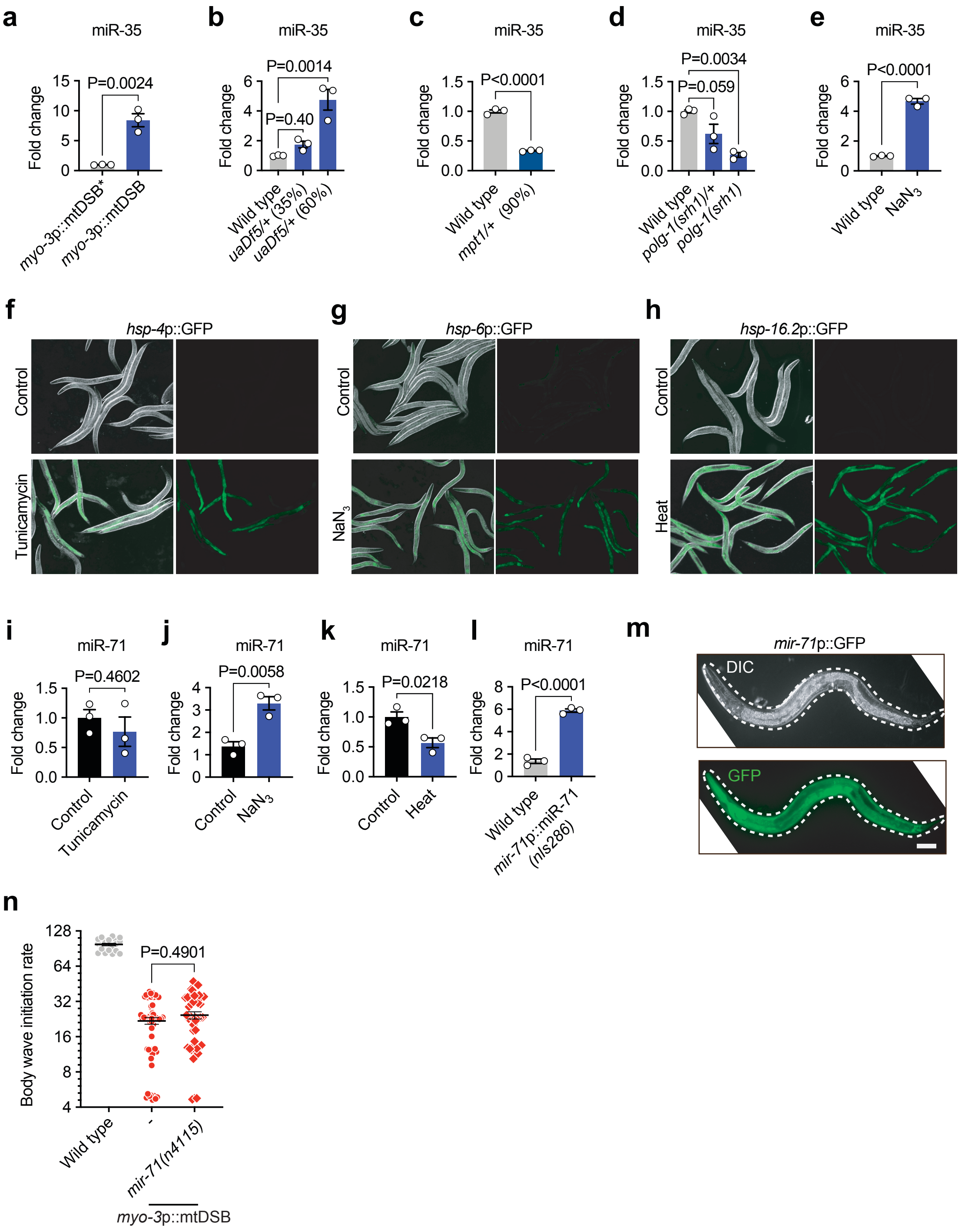

Extended Data Fig. 1. Mitochondrial stress up-regulates miR-35 family and miR-71 miRNAs.

a-e, qPCR analysis of miR-35 levels. Columns represent mean ± SEM; *n* = 3; (a), (c), and (e) two-way Student's t test; (b) and (d) one-way ANOVA with Tukey's post hoc test. **f-h**, representative photomicrographs of animals expressing (f) *hsp-4*::GFP, (g) *hsp-6p*::GFP, and (h) *hsp-16.2p*::GFP under either control or specific stressor conditions. Tunicamycin, 0.02 µg/ml; sodium azide (NaN_3), 1 mM. For the heat stress, L4 animals were transferred to 37°C

for 1 h, followed by 5 h recovery at 20°C. **i-l**, qPCR analysis of miR-71 levels. Columns represent mean ± SEM; n = 3; two-way Student's t test. **M**, representative photomicrograph of an animal (outlined by a white dashed line) expressing *mir-71p*::GFP. Scale bar, 50 µm. **n**, quantification of body wave initiation rate (using WormLab automated software analysis) of L4 animals placed in liquid. Bars represent mean ± SEM; n = 21, 56 and 45; one-way ANOVA with Tukey's post hoc test. Source data are provided as a Source Data file.

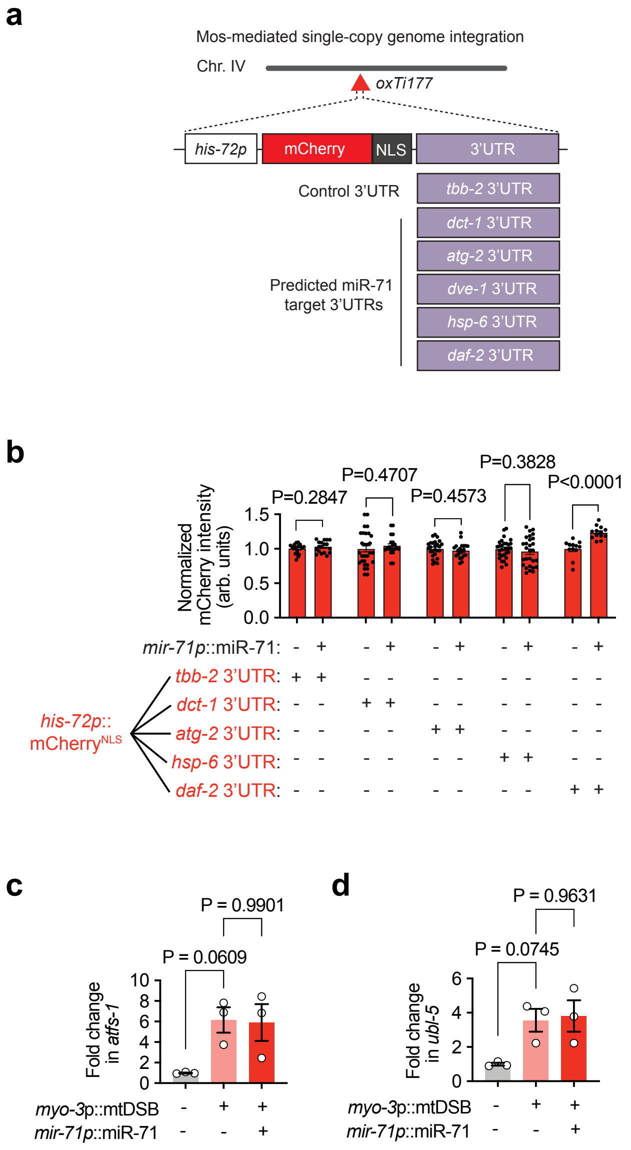

Extended Data Fig. 2. Screening of predicted miR-71 targets reveals that *dve-1* is a specific target of miR-71.

a, schematic diagrams and chromosomal integration site of 3'UTR reporters of predicted miR-71 targets. **b**, quantification of mCherry fluorescence intensity of the heads of animals expressing the 3'UTR reporter. Columns represent mean ± SEM; two-way Student's t test. **c**, and **d**, qPCR analysis of *atfs-1* and *ubl-5* transcript levels, respectively. Columns represent mean ± SEM; $n = 3$; one-way ANOVA with Tukey's post hoc test. Source data are provided as a Source Data file.

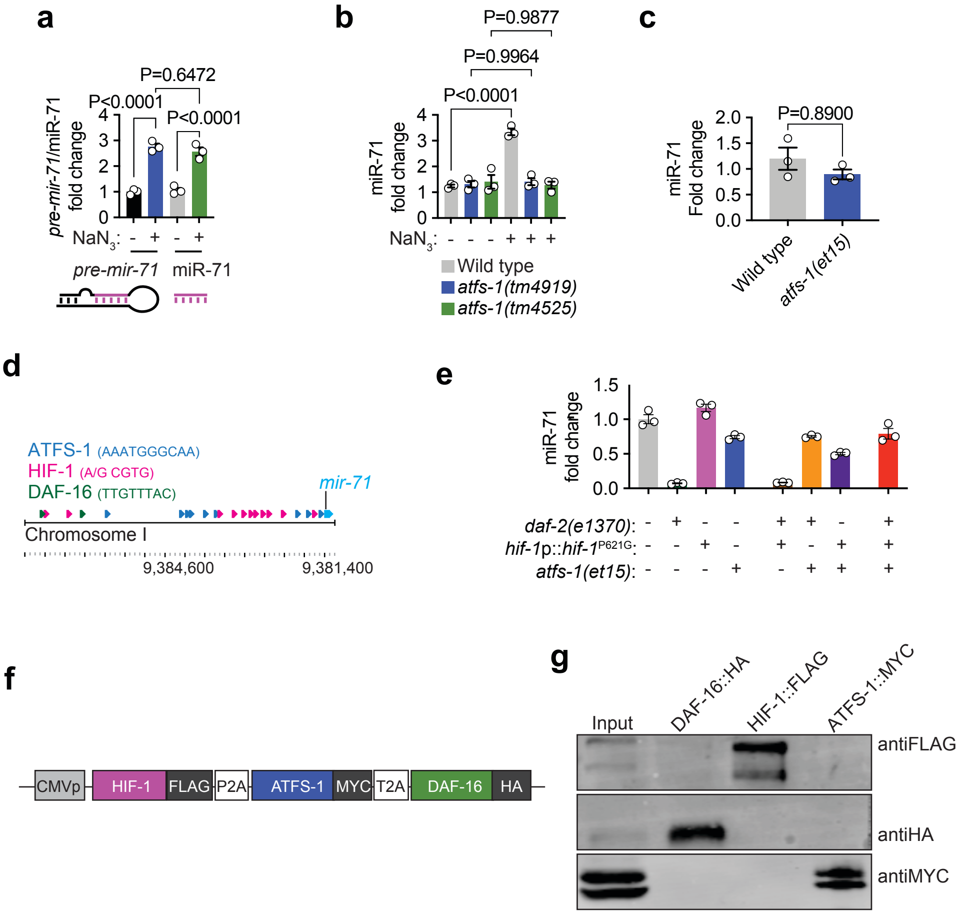

Extended Data Fig. 3. ATFS-1, HIF-1, and DAF-16 regulate miR-71 transcription.

a, qPCR analysis of *pre-mir-71* and mature miR-71 levels. Columns represent mean ± SEM; *n* = 3; one-way ANOVA with Tukey's post hoc test. NaN_3, sodium azide. **b-c**, qPCR analysis of miR-71 levels. Columns represent mean ± SEM; *n* = 3; (b) one-way ANOVA with Tukey's post hoc test and (c) two-way Student's t test. **d**, schematic diagram of putative ATFS-1, HIF-1, and DAF-16 binding motifs upstream of the *mir-71* gene. **e**, qPCR analysis of miR-71 levels. Columns represent mean ± SEM; *n* = 3. **f**, schematic diagram of the mammalian cell culture construct to express HIF-1, ATFS-1, and DAF-16 simultaneously. **g**, representative western blot of coimmunoprecipitation experiments show no physical interaction between DAF-16, HIF-1, and ATFS-1. Source data are provided as a Source Data file.

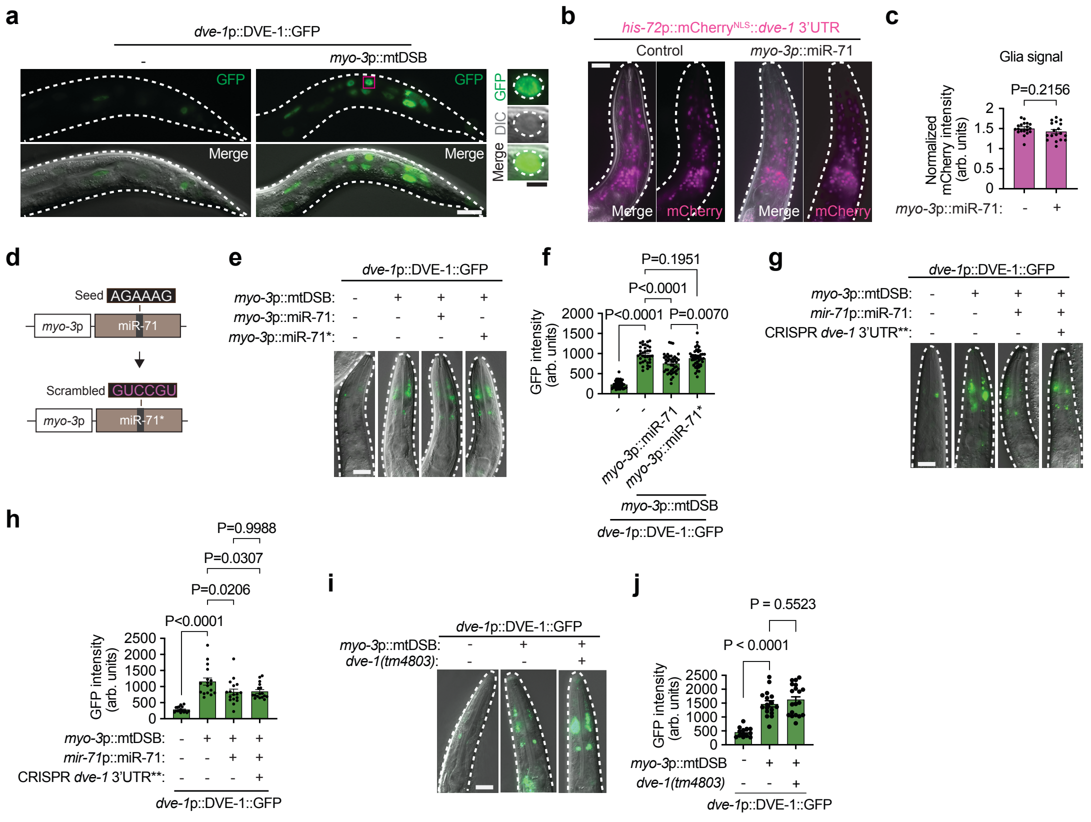

Extended Data Fig. 4. miR-71 regulates cell-non-autonomous mitochondrial stress signaling.

a, representative photomicrographs of the posterior of animals (outlined by a white dashed line) expressing *dve-1p*::DVE-1::GFP. Scale bar, 20 μm. Red box indicates area enlarged on panels on the right which show GFP signal in the nucleus of an intestinal cell. Scale bar, 5 μm.

b and **c,** (b) representative photomicrographs of the heads of animals (outlined by a white dashed line) expressing *dve-1p* 3'UTR reporter and (c) quantification of mCherry fluorescence intensity. Columns represent mean ± SEM; for b, n = 18, 18, 29, 22, 26, 21, 29, 28, 12, and 13, for c, n = 19 and 18; one-way ANOVA with Tukey's post hoc test. Scale bar, 20 μm. **d**, schematic diagram of the seed sequence of miR-71 and its scrambling in the muscle-specific overexpression transgene. **e-j**, (e), (g), and (i) representative photomicrographs of the heads of animals (outlined by a white dashed line) expressing *dve-1p*::DVE-1::GFP. (f), (h), and (j) quantification of GFP fluorescence intensity. Columns represent mean ± SEM; for f, n = 42, 34, 43 and 50, for h, n = 17; for j, n = 13, 17 and 19; one-way ANOVA with Tukey's post hoc test. Scale bar, 20 μm. Source data are provided as a Source Data file.

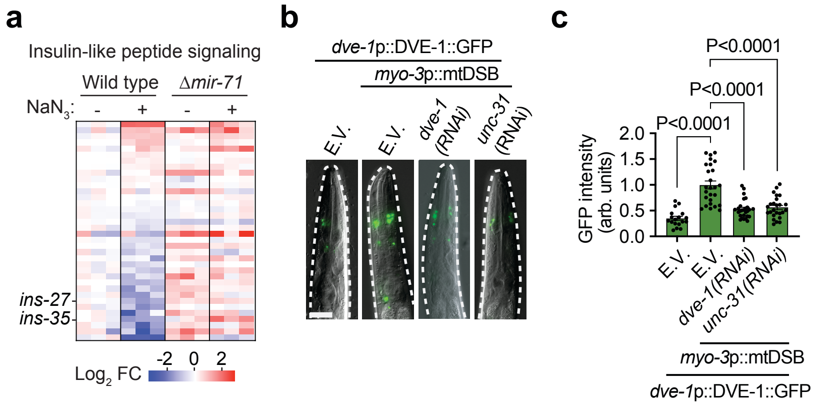

Extended Data Fig. 5. ***unc-31*** **is required for DVE-1 activation in glia upon muscle-specific mtDSB.**

a, heat map of RNA-seq results for transcripts involved in insulin-like peptide signaling. $n = 3$. **b**, representative photomicrographs of the heads of animals (outlined by a white dashed line) expressing *dve-1p*::DVE-1::GFP. **c**, Quantification of GFP fluorescence intensity. Columns represent mean ± SEM; n = 26, 18, 27 and 25; one-way ANOVA with Tukey's post hoc test. Scale bar, 20μm. Source data are provided as a Source Data file.

Extended Figure 3g original blots

Anti HA (detecting DAF-16)

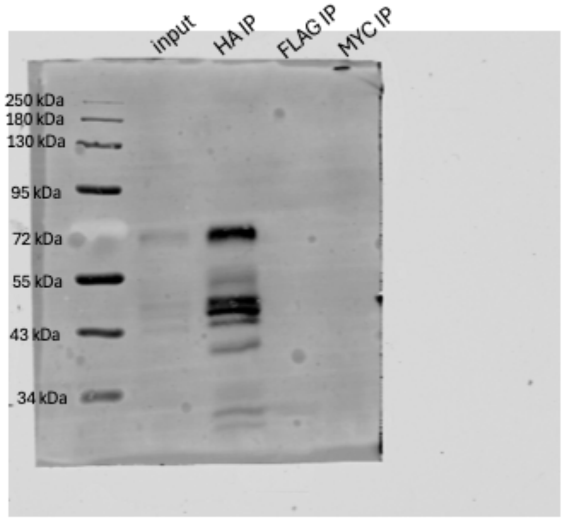

Anti MYC (detecting ATFS-1)

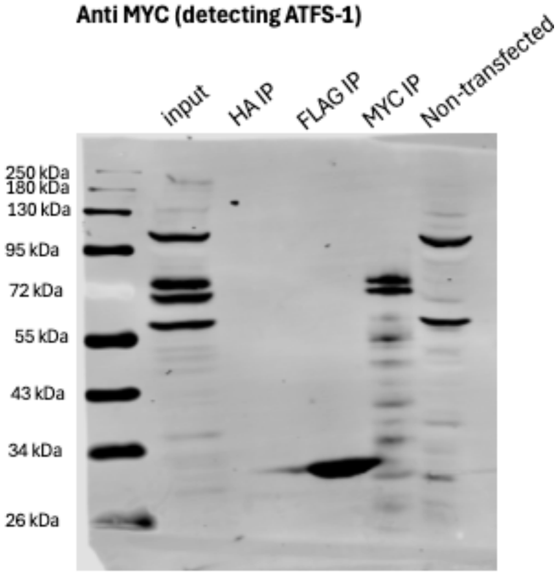

Anti FLAG (detecting HIF-1)

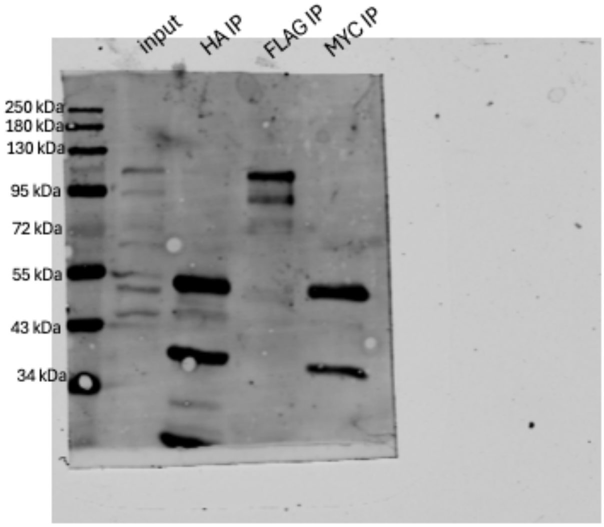

Supplement: Supplementary file 1 — Supplementary Information [file 41467_2025_67198_MOESM1_ESM.pdf]
