# Supplementary material for: The microRNA miR-71 suppresses maladaptive UPRmt signaling through both cell-autonomous and cell-non-autonomous mechanisms
Source: Nat Commun. 2025 Dec 14;17:510. doi: 10.1038/s41467-025-67198-2 (PMC12804905; doi:10.1038/s41467-025-67198-2)
Supplement: Supplementary file 2 — Description of Additional Supplementary Files [file 41467_2025_67198_MOESM2_ESM.pdf]

Description of Additional Supplementary Files

File Name: Supplementary Data 1
Description: small RNA levels in animals with a catalytically active or inactive transgene introduced to cause mtDNA double-stranded breaks. A raw count table of selected sequences for each sample was generated for edgeR to perform the differential expression analysis between each group. EdgeR used a trimmed mean of M-values to compute scale factors for library size normalization and used the Cox-Reid profile-adjusted likelihood method to estimate dispersions and the quasi-likelihood F-test to determine differential expression (two-sided comparison).

File Name: Supplementary Data 2
Description: Significantly differentially expressed genes (FDR<0.05) in wild-type animal vs miR-71 knock-out animal with and without azide stress. Sequencing was carried out using a 2x150bp paired-end (PE) configuration; image analysis and base calling were conducted by the HiSeq Control Software (HCS) + OLB + GAPipeline-1.6 (Illumina) on the HiSeq instrument. The sequences were processed and analyzed by GENEWIZ (two-sided comparisons).

File Name: Supplementary Data 3
Description: list of genes knocked down to find signalling molecule in muscle-to-glia stress signalling

File Name: Supplementary Data 4
Description: C. elegans strains used for this study

File Name: Supplementary Data 5
Description: plasmids used for this study

File Name: Supplementary Data 6
Description: primers used for this study
