# Supplementary material for: The microRNA miR-71 suppresses maladaptive UPRmt signaling through both cell-autonomous and cell-non-autonomous mechanisms
Source: Nat Commun. 2025 Dec 14;17:510. doi: 10.1038/s41467-025-67198-2 (PMC12804905; doi:10.1038/s41467-025-67198-2)
Supplement: Supplementary file 4 — Reporting Summary [file 41467_2025_67198_MOESM4_ESM.pdf]

nature portfolio

Corresponding author(s): Steven Zuryn

Last updated by author(s): 31/10/2025

Reporting Summary

Nature Portfolio wishes to improve the reproducibility of the work that we publish. This form provides structure for consistency and transparency in reporting. For further information on Nature Portfolio policies, see our Editorial Policies and the Editorial Policy Checklist.

Statistics

For all statistical analyses, confirm that the following items are present in the figure legend, table legend, main text, or Methods section.

n/a	Confirmed	
☐	☒	The exact sample size (*n*) for each experimental group/condition, given as a discrete number and unit of measurement
☐	☒	A statement on whether measurements were taken from distinct samples or whether the same sample was measured repeatedly
☐	☒	The statistical test(s) used AND whether they are one- or two-sided<br>*Only common tests should be described solely by name; describe more complex techniques in the Methods section.*
☐	☒	A description of all covariates tested
☒	☐	A description of any assumptions or corrections, such as tests of normality and adjustment for multiple comparisons
☐	☒	A full description of the statistical parameters including central tendency (e.g. means) or other basic estimates (e.g. regression coefficient) AND variation (e.g. standard deviation) or associated estimates of uncertainty (e.g. confidence intervals)
☐	☒	For null hypothesis testing, the test statistic (e.g. *F*, *t*, *r*) with confidence intervals, effect sizes, degrees of freedom and *P* value noted<br>*Give P values as exact values whenever suitable.*
☒	☐	For Bayesian analysis, information on the choice of priors and Markov chain Monte Carlo settings
☒	☐	For hierarchical and complex designs, identification of the appropriate level for tests and full reporting of outcomes
☒	☐	Estimates of effect sizes (e.g. Cohen's *d*, Pearson's *r*), indicating how they were calculated

Our web collection on statistics for biologists contains articles on many of the points above.

Software and code

Policy information about availability of computer code

Data collection	The obtained reads for small RNA sequencing were mapped to the C. elegans genome version ce10 and annotated to miRBase release 21. For mRNA sequencing, in order to remove technical sequences, including adapters, PCR primers, or fragments thereof, and quality of bases lower than 20, pass filter data in FASTQ format were processed by Cutadapt (V1.9.1, phred cutoff: 20, error rate: 0.1, adapter overlap: 1bp, min. length: 75, proportion of N: 0.1) to filter reads. The C. elegans reference genome sequence and gene model annotation files were downloaded from https://www.ncbi.nlm.nih.gov/assembly/GCF_000002985.6/. Hisat2 (v2.0.1) was used to index the reference genome sequence and to align filtered reads to the reference genome. FASTA format transcripts were converted from the known gff annotation file and indexed properly. Then, with the file as a reference gene file, HTSeq (v0.6.1) estimated gene and isoform expression levels from the pair-end filtered data. Differential gene expression analysis was completed using the DESeq2 Bioconductor package, with an adjusted P value cutoff of P < 0.05 for differentially expressed genes.
Data analysis	GraphPad Prism (version 10.2.0)

For manuscripts utilizing custom algorithms or software that are central to the research but not yet described in published literature, software must be made available to editors and reviewers. We strongly encourage code deposition in a community repository (e.g. GitHub). See the Nature Portfolio guidelines for submitting code & software for further information.

Data

Policy information about availability of data

All manuscripts must include a data availability statement. This statement should provide the following information, where applicable:

- Accession codes, unique identifiers, or web links for publicly available datasets
- A description of any restrictions on data availability
- For clinical datasets or third party data, please ensure that the statement adheres to our policy

Original data used in each main and supplementary figure has been provided in the data source file. Additional graphical and tabulated supplementary material has been provided in the supplementary documents. This paper analyzes existing, publicly available data. The accession numbers for these datasets are listed in the Materials and Methods section. RNA sequencing data generated by this study can be found at doi.org/10.5281/zenodo.17479944. Small RNA sequencing has been deposited to NCBI Sequence Read Archive, accession number PRJNA1354193.

Research involving human participants, their data, or biological material

Policy information about studies with human participants or human data. See also policy information about sex, gender (identity/presentation), and sexual orientation and race, ethnicity and racism.

Reporting on sex and gender	*Use the terms sex (biological attribute) and gender (shaped by social and cultural circumstances) carefully in order to avoid confusing both terms. Indicate if findings apply to only one sex or gender; describe whether sex and gender were considered in study design; whether sex and/or gender was determined based on self-reporting or assigned and methods used.* *Provide in the source data disaggregated sex and gender data, where this information has been collected, and if consent has been obtained for sharing of individual-level data; provide overall numbers in this Reporting Summary. Please state if this information has not been collected.* *Report sex- and gender-based analyses where performed, justify reasons for lack of sex- and gender-based analysis.*
Reporting on race, ethnicity, or other socially relevant groupings	*Please specify the socially constructed or socially relevant categorization variable(s) used in your manuscript and explain why they were used. Please note that such variables should not be used as proxies for other socially constructed/relevant variables (for example, race or ethnicity should not be used as a proxy for socioeconomic status).* *Provide clear definitions of the relevant terms used, how they were provided (by the participants/respondents, the researchers, or third parties), and the method(s) used to classify people into the different categories (e.g. self-report, census or administrative data, social media data, etc.)* *Please provide details about how you controlled for confounding variables in your analyses.*
Population characteristics	*Describe the covariate-relevant population characteristics of the human research participants (e.g. age, genotypic information, past and current diagnosis and treatment categories). If you filled out the behavioural & social sciences study design questions and have nothing to add here, write "See above."*
Recruitment	*Describe how participants were recruited. Outline any potential self-selection bias or other biases that may be present and how these are likely to impact results.*
Ethics oversight	*Identify the organization(s) that approved the study protocol.*

Note that full information on the approval of the study protocol must also be provided in the manuscript.

Field-specific reporting

Please select the one below that is the best fit for your research. If you are not sure, read the appropriate sections before making your selection.

☒ Life sciences ☐ Behavioural & social sciences ☐ Ecological, evolutionary & environmental sciences

For a reference copy of the document with all sections, see nature.com/documents/nr-reporting-summary-flat.pdf

Life sciences study design

All studies must disclose on these points even when the disclosure is negative.

Sample size	No sample size calculations were performed. At least 20 animals were used for locomotion assays for each condition. By previously performing this assay many times, we find that 20 animals is sufficient to detect reliable statistical differences among different genetic backgrounds and treatment conditions. For immunoblots, co-immunoprecipitations and qPCRs, we performed n = or > 3 biological replicates. n = 3 is a widely used and accepted n value for these types of experiments in this field and produces reliable and sufficient statistical differences among different genetic backgrounds and treatment conditions. In instances where more samples were readily available, we used n >3. For imaging related quantification, we used n > 10. n values corresponding to each figure are shown in the figure itself or figure legend. By previously performing imaging experiments many times, we find that 10 animals is sufficient to detect reliable statistical differences among different genetic backgrounds and treatment conditions.
Data exclusions	For aged animals locomotion assay, dead worms, bagging worms and bursted animals were excluded. for qPCR, raw CT value in each sample with a difference 0.5 or greater from other two technical replicates was excluded.

Replication	All locomotion assays were repeated independently at least twice.<br>All immunoblots were repeated independently at least three times.<br>All qPCR results had at least three biological replicates and each sample had three technical replicates.<br>All small RNA and RNA sequencing experiments were repeated three independent times.<br>All the replication attempts were successful.<br>For microscopy, at least 10 independent animals were imaged.
Randomization	For C. elegans experiments, populations of animals were raised under identical conditions. Worms were randomly picked from populations for qPCR related assays, locomotion assays, and imaging. For cell culture related assays, cells were grown on tissue culture dishes and were scraped down for assays.
Blinding	Experimenters were not blinded to strain or genotype. Individual animals were picked or washed off plates randomly for each assay.

Reporting for specific materials, systems and methods

We require information from authors about some types of materials, experimental systems and methods used in many studies. Here, indicate whether each material, system or method listed is relevant to your study. If you are not sure if a list item applies to your research, read the appropriate section before selecting a response.

Materials & experimental systems

n/a	Involved in the study
☐	☒ Antibodies
☐	☒ Eukaryotic cell lines
☒	☐ Palaeontology and archaeology
☐	☒ Animals and other organisms
☒	☐ Clinical data
☒	☐ Dual use research of concern
☒	☐ Plants

Methods

n/a	Involved in the study
☒	☐ ChIP-seq
☒	☐ Flow cytometry
☒	☐ MRI-based neuroimaging

Antibodies

Antibodies used	The antibodies and dilutions used in this study were: anti-HA (Cell Signalling, Cat. 3724), 1:5,000; anti-FLAG (Sigma-Aldrich, Cat. F3165), 1:5,000; anti-Myc (Invitrogen, Cat. PA1-981), 1:5,000; IRDye 680RD goat anti-mouse IgG (LI-COR, Cat. 68070), 1:20,000; and IRDye 800CW goat anti-rabbit IgG (LI-COR, Cat. 32211), 1:20,000.
Validation	All antibodies used in this study are commercially available and have been validated by the manufacturer.<br>Anti-HA (clone. c29F4), applications: Western Blot, Immunoprecipitation, Immunohistochemistry, ChIP, CUT&RUN, CUT&Tag, Dot Blot, eCLIP, Immunofluorescence, Flow Cytometry. Species Reactivity: All Species Expected. Manufacturer's website: https://www.cellsignal.com/products/primary-antibodies/ha-tag-c29f4-rabbit-mab/3724<br>Anti-FLAG (clone. M2), applications: Immunoblotting, Immunoprecipitation, Immunocytochemistry, Immunofluorescence, ELISA, EIA, ChIP, Electron Microscopy, Flow Cytometry, Supershift assays. Species Reactivity: All Species. Manufacturer's website: https://www.sigmaaldrich.com/AU/en/product/sigma/f3165<br>Anti-Myc, applications: Western Blot, Immunocytochemistry, ChIP, Immunohistochemistry, Immunoprecipitation, Flow Cytometry. Species Reactivity: Tag. Manufacture's statement: This Antibody was verified by Relative expression to ensure that the antibody binds to the antigen stated. Manufacturer's website: https://www.thermofisher.com/antibody/product/Myc-Tag-Antibody-Polyclonal/PA1-981?gclid=CjwKCAjwpuajBhBpEiwA_Ztfhf2e-gjJ8SsK6cdWGCcxdpfXn9e2KlRqNPhNf7aBSyP6ERx4HIad6RoCvkMQAvD_BwE&ef_id=CjwKCAjwpuajBhBpEiwA_Ztfhf2e-gjJ8SsK6cdWGCcxdpfXn9e2KlRqNPhNf7aBSyP6ERx4HIad6RoCvkMQAvD_BwE:G:s&s_kwcid=AL!3652!3!459737518508!!!g!!10950825775!106531320406&cid=bid_pca_aup_r01_co_cp1359_pjt0000_bid00000_0se_gaw_dy_pur_con

Eukaryotic cell lines

Policy information about cell lines and Sex and Gender in Research

Cell line source(s)	HEK 293T/17 is from ATCC [CRL11268] and CAL51 was obtained from DSMZ
Authentication	Cell lines were authenticated by STR profiling
Mycoplasma contamination	PCR analysis confirming negative for mycoplasma contamination
Commonly misidentified lines<br>(See ICLAC register)	No commonly misidentified cell lines were used

Animals and other research organisms

Policy information about studies involving animals; ARRIVE guidelines recommended for reporting animal research, and Sex and Gender in Research

Laboratory animals	Caenorhabditis elegans, hermaphrodite, age L1-7 days old (wild type N2 Bristol, mutants and transgenic animals). All mutants and transgenic animals described in this study are in N2 Bristol background.<br>N2 Bristol wild type<br>polg-1(srh1)/mnC1 [dpy-10(e128) unc-52(e444) nIs190(myo-2::GFP) let-?] II<br>CL2070 dvIs70[hsp-16.2p::GFP + rol-6(su1006)]<br>QC115 atfs-1(et15)<br>atfs-1(cmh15) V<br>TM4525 atfs-1(tm4525)<br>TM4919 atfs-1(tm4919)/nT1 [qIs51]<br>SJ4005 zcIs4 [hsp-4p::gfp] V<br>SJ4100 zcIs13 [hsp-6p::GFP] V<br>SJZ540 uaDf5/+<br>mpt1 mpt-1/+ (also called mptDf1/+)<br>MT12993 mir-71(n4115) I.<br>SJZ732 miR-71(n4115) I ; foxsi1 [myo-3p::MTS::Pst1::mkate2::DD::SL2::His58::GFP+unc-119(+)unc119(ed3)]] III<br>SJZ1121 foxsi176 [his-72p::mCherry::2xNLS::dve-1 3'UTR] IV; miR-71 (n4115) I<br>SJZ8 foxsi1 [myo-3p::MTS::Pst1::mKate2::DD::SL2::his-58::GFP + unc-119(+)] unc-119(ed3) III<br>SJZ1360 foxSi212 [myo-3p::miR-71] IV<br>SJZ1378 foxSi215 [miR-71p::miR-71] IV<br>SJZ1086 foxsi176 [his-72p::mCherry::2xNLS::dve-1 3'UTR] IV<br>SJZ1122 foxsi185 [his-72p::mCherry::2xNLS::dve-1 3'UTR**] IV<br>SJZ41 foxsi1 [myo-3p::MTS::Pst1::mKate2::DD::SL2::his-58::GFP + unc-119(+)]; atfs-1(tm4525) V<br>SJZ86 zcIs13 [hsp-6::GFP] V; foxEx10[myo-3pr::MTS::PstI::mKate2::DD+Odr-1pr::DsRed (10ng/ul +50ng/ul)]<br>SJZ87 zcIs9 [hsp-60::GFP + lin-15(+)] V; foxEx10[myo-3pr::MTS::PstI::mKate2::DD+Odr-1pr::DsRed (10ng/ul +50ng/ul)]<br>SJZ21 foxSi1 [myo-3pr::MTS::PstI::mKate2::DD::SL2::his58::GFP] III; atfs-1 (tm4525) V<br>SJZ344 foxSi74 [myo-3p::ΔMTS2-34ATFS-1::Cerulean] I; foxSi1 [myo-3p::MTS::PstI::mKate2::DD::SL2::His58::GFP]III; atfs-1(tm4525) V<br>SJZ1114 foxSi74 [myo-3p::ΔMTS2-34ATFS-1::Cerulean] I; foxSi1 [myo-3p::MTS::PstI::mKate2::DD::SL2::His58::GFP]III; atfs-1(tm4525) V; nIs286 [mir-71(+) + sur-5::GFP] X<br>SJZ1288 miR-71(n4115) I; ZcIs39[dve-1p::dve-1::GFP] II; foxEx308 [myo-3p::mtPstI::mKate2::tbb-2 3'UTR + odr-1p::DsRed]<br>SJZ546 nIs286 [mir-71(+) + sur-5::GFP] X; foxsi1[myo-3p::MTS::Pst1::mKate2::DD::SL2::his-58::GFP + unc-119(+)] unc-119(ed3) III<br>SJZ1377 foxSi214 [rgef-1p::miR-71] IV<br>SJZ1361 foxSi213 [ges-1p::miR-71] IV<br>SJZ1098 foxEx285 [myo3p::MTS::Pst1::mkate2::tbb2 3'UTR]; daf-16(mu86) I; muIs61 [(pKL78) daf-16::GFP+rol-6 (su1006)]<br>SJZ1076 foxEx282 [myo3p::MTS::Pst1::mkate2::tbb2 3'UTR]; OpIS206 [hif-1p::hif-1::GFP::hif-1 3'UTR]<br>SJZ165 foxEx44 [ATFS-1p::atfs-1::GFP + odr-1p::DsRed (2ng/ul+50ng/ul)]; foxSi19 [myo-3p::PstI::mKate2::DD::3'UTR] III<br>CF1038 daf-16(mu86) I<br>ZG596 hif-1(ia7) V<br>SJZ1094 atfs-1(tm4525) V; daf-16(mu86) I<br>SJZ1237 ZcIs39 [dve-1p::dve-1::GFP]; foxEx305 [myo-3p::mtPstI::tbb-2 3'UTR]<br>SJ4197 ZcIs39 [dve-1p::dve-1::GFP] II<br>SJZ1362 foxEx321 [mir-228p::mkate2::tbb2-3'UTR]; foxsi14 [myo-3p::MTS::Pst1::mkate2::tbb-2 3'UTR]III; zcIs39 [dve-1p::dve-1::GFP] II<br>SJZ1288 miR-71(n4115) I; ZcIs39 [dve-1p::dve-1::GFP] II; foxEx308 [myo-3p::mtPstI::mKate2::tbb-2 3'UTR + odr-1p::DsRed]<br>SJZ1808 ZcIs39 [dve-1p::dve-1::GFP::unc-54 3'UTR]; miR-71 (n4115)<br>SJZ1426 foxsi212 [myo-3p::miR-71] IV; zcIs39 [dve-1::dve-1::GFP]; foxEx308 [myo-3p::mtPstI::mkate2::tbb-2 3'UTR]<br>SJZ1407 foxSi215 [miR-71p::miR-71] IV; ZcIs39 [dve-1p::dve-1::GFP]; foxEx308 [myo-3p::mtPstI::mKate2::tbb-2 3'UTR + odr-1p::DsRed]<br>SJZ1062 foxsi161 [his-72p::mCherry::2xNLS::tbb-2 3'UTR] IV<br>SJZ1084 foxsi174 [his-72p::mCherry::2xNLS::atg-2 3'UTR] IV<br>SJZ1085 foxsi175 [his-72p::mCherry::2xNLS::dct-1 3'UTR] IV<br>SJZ1117 foxsi183 [his-72p::mCherry::2xNLS::daf-2 3'UTR] IV<br>SJZ1118 foxsi184 [his-72p::mCherry::2xNLS::hsp-6 3'UTR] IV<br>SJZ1086 foxsi176 [his-72p::mCherry::2xNLS::dve-1 3'UTR] IV<br>SJZ1121 foxsi176 [his-72p::mCherry::2xNLS::dve-1 3'UTR] IV; miR-71 (n4115) I<br>SJZ1575 foxsi230 [myo-3p::miR-71**] IV; zcIs39 [dve-1::dve-1::GFP]; foxEx308 [myo-3p::mtPstI::mkate2::tbb-2 3'UTR]<br>SJZ1426 foxsi212 [myo-3p::miR-71] IV; zcIs39 [dve-1::dve-1::GFP]; foxEx308 [myo-3p::mtPstI::mkate2::tbb-2 3'UTR]<br>SJZ1788 foxEx308 [myo-3p::mtPstI::mKate2::tbb-2 3'UTR + odr-1p::DsRed] ; ZcIs39 [dve-1p::dve-1::GFP::unc-54 3'UTR]; syb3514 [dve-1 3'UTR**]; foxSi215 [miR-71p::miR-71] IV<br>SJZ1407 foxSi215 [miR-71p::miR-71] IV; ZcIs39 [dve-1p::dve-1::GFP]; foxEx308 [myo-3p::mtPstI::mKate2::tbb-2 3'UTR + odr-1p::DsRed]
Wild animals	This study did not involve wild animals

Reporting on sex	All assay were performed on hermaphrodite C. elegans
Field-collected samples	This study did not involve samples collected from the field
Ethics oversight	No ethics approval is required for this study

Note that full information on the approval of the study protocol must also be provided in the manuscript.

Plants

Seed stocks	*Report on the source of all seed stocks or other plant material used. If applicable, state the seed stock centre and catalogue number. If plant specimens were collected from the field, describe the collection location, date and sampling procedures.*
Novel plant genotypes	*Describe the methods by which all novel plant genotypes were produced. This includes those generated by transgenic approaches, gene editing, chemical/radiation-based mutagenesis and hybridization. For transgenic lines, describe the transformation method, the number of independent lines analyzed and the generation upon which experiments were performed. For gene-edited lines, describe the editor used, the endogenous sequence targeted for editing, the targeting guide RNA sequence (if applicable) and how the editor was applied.*
Authentication	*Describe any authentication procedures for each seed stock used or novel genotype generated. Describe any experiments used to assess the effect of a mutation and, where applicable, how potential secondary effects (e.g. second site T-DNA insertions, mosiacism, off-target gene editing) were examined.*
